# Tissue sublimation follow transarterial embolization of a follicular nodular hyperplasia of the liver—report of a case

**DOI:** 10.1186/s12876-017-0648-z

**Published:** 2017-08-01

**Authors:** Peter C. Ambe, Stefan Jansen, Hubert Zirngibl

**Affiliations:** Department of Surgery, Helios University Hospital Wuppertal, Witten – Herdecke University, Heusnerstr. 40, 42283 Wuppertal, Germany

**Keywords:** Follicular nodular hyperplasia, Transarterial embolization, Tissue sublimation, Cholecystitis

## Abstract

**Background:**

Follicular nodular hyperplasia (FNH) is a common benign liver tumor for which conservative management is indicated. Surgical or interventional management is indicated in symptomatic cases. Transarterial embolization (TAE) has been extensively used to manage unresectable liver tumors. Sublimation describes a change of physical state from solid to gas. Hepatic tissue sublimation following TAE has so far not been reported in medical literature.

**Case presentation:**

A 30 year - old male patient presenting with pain to the upper abdomen due to a large FNH was managed with TAE. Routine radiographic control on post-intervention day one was within normal limits. Imaging due to right upper quadrant pain with fever and elevated inflammatory markers and liver enzymes on day two after TAE revealed a marked reduction of the FNH accompanied by the presence of a large volume of gas collection without signs of abscess formation. This change of state from solid to gas without sign of abscess formation within 2 days after TAE was described as hepatic tissue sublimation. The patient was managed conservatively and discharge 12 days after TAE.

**Conclusion:**

Tissue sublimation has hardly been reported in medical literature. This to the best of our knowledge is the first documented case of hepatic tissue sublimation following TAE.

## Background

Follicular nodular hyperplasia (FNH) [1, 2] is a benign lesion of the liver that is often incidentally discovered during abdominal imaging [1]. FNH has no malignant potential and is most commonly seen in female patients of reproductive age, a fact that has been associated with the use of oral contraceptives [3]. Although FNH is mostly clinically inapparent, rapid growth might lead to symptoms, especially upper abdominal pain. Patients with rapid progressing FNH therefore are usually candidates for surgical referral. Resection of the lesion is indicated to release symptoms and to exclude the presence of hepatic adenoma, a precancerous lesion with morphologic similarities to FNH.

Besides surgical resection, less invasive interventional techniques have been used to manage liver tumors [4]. A major advantage of less invasive techniques is the low risk of complication in comparison to the risk of complication following surgical resection. Transarterial embolization (TAE) represents a standard interventional technique for the management of hepatic lesions [5]. Hepatic tissue sublimation (change of physical state from solid to gas) has so far not been reported as a sequela of TAE. This to the best of our knowledge is the first report of tissue sublimation following TAE.

## Case presentation

**Fig. 1:**
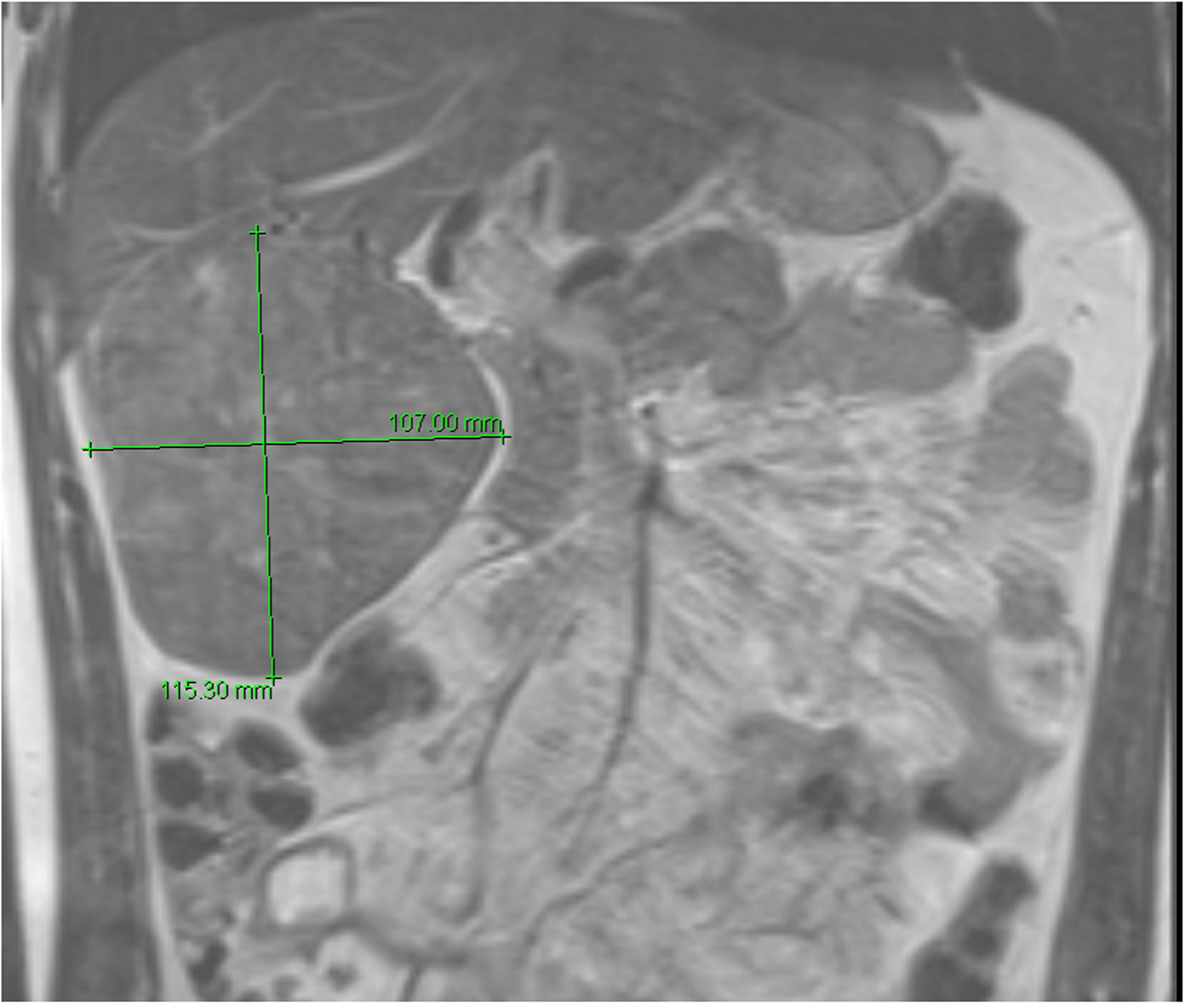
T1 weighted MRI showing a 10 × 11 cm mass in the right liver lobe

**Fig. 2:**
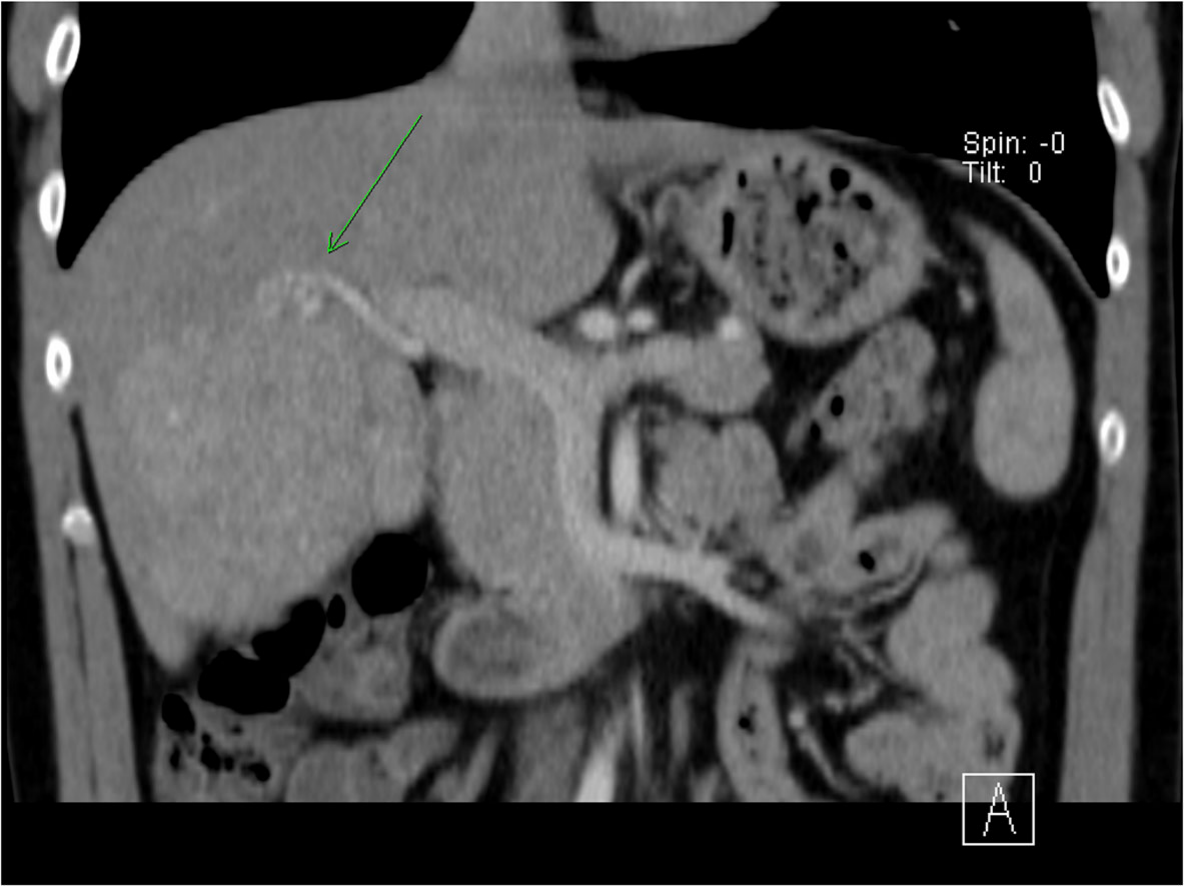
Ct scan. Note the close proximity to the central hepatic vasculature (*arrow*)

**Fig. 3:**
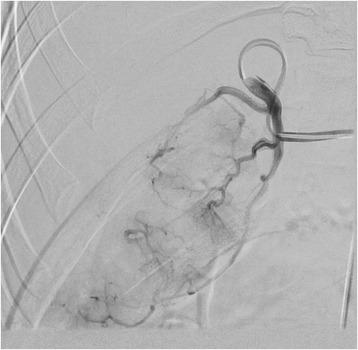
Transarterial embolization

**Fig. 4:**
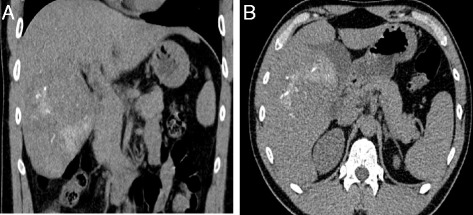
**a** & **b** Ct scan on day one following TAE

**Fig. 5:**
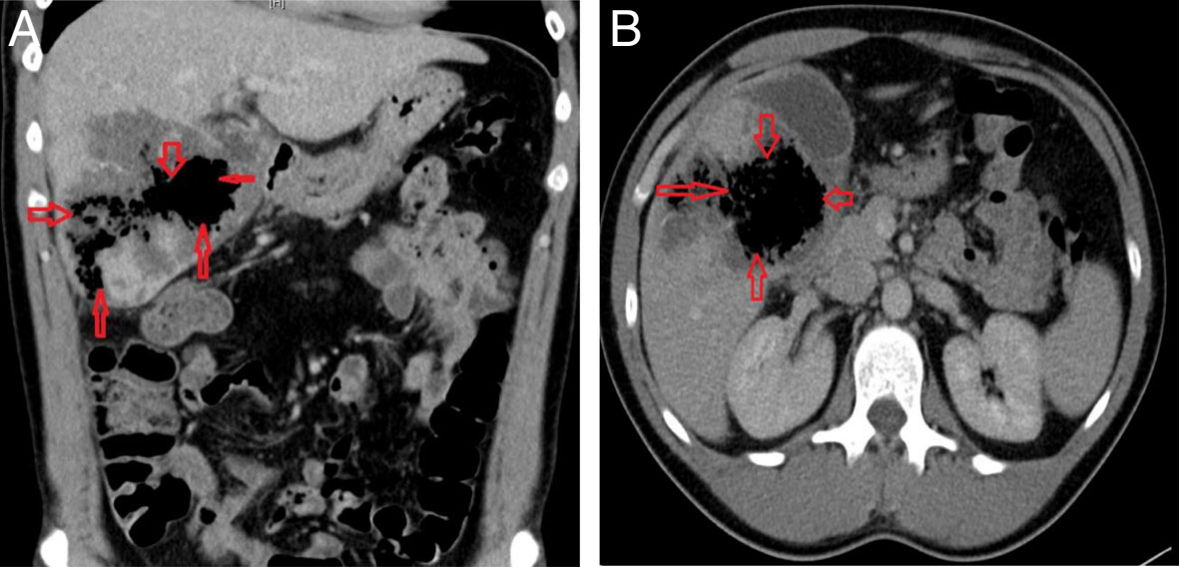
**a** & **b** Ct scan on day two after TAE. Note the gas collection (*red arrows*) without signs of abscess formation

**Fig. 6:**
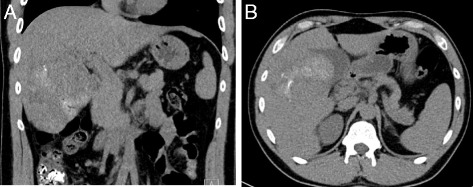
**a** & **b**: Ct scan 3 weeks after TAE showing a complete gas resorption

A 30 year-old male patient was referred to our tertiary surgical department with progressive pain to the upper abdomen. A hepatic mass was diagnosed 9 months previously. Fine needle biopsy confirmed the mass to be an FNH. At that time “watchful waiting” was recommended. A mass could be palpated in the right upper quadrant during physical examination. Blood chemistry including liver enzymes were within normal limits. A large mass was seen in the right liver lobe during ultrasound (US) [6]. Magnetic resonance imaging (MRI) confirmed a 10 × 11 cm mass in the right liver lobe (Fig. 1) with a significant increase in size compared to the MRI findings about 9 months earlier. A contrast enhanced computed tomography (ct) with angiography was ordered to better study the proximity of the mass to the large liver vessels (Fig. 2). The case was discussed at the interdisciplinary board. The risk of complications following surgical resection was deemed high due to the proximity of the mass to the central hepatic vessels. TAE was favored. TAE was performed by an experienced interventional radiologist using Embazene® microspheres as reported elsewhere [7–9] (Fig. 3). Routine Ct on post-interventional day one was within normal limits (Fig. 4a & b). The patient developed pain to the right upper quadrant on day two following TAE with fever (39 °C axillar). The right upper quadrant was tender on palpation. Blood chemistry revealed elevated liver enzymes: bilirubin 1.22 mg/dl (normal limit: 1.2 mg/dl), GOT: 1692 U /l (normal limits 10–50 U/L), GPT: 1569 (normal limits: 10–50 U/l), GGT 165 (normal limits: 0–66 U/l), AP 558 (normal limits: 0–98 U /l). Inflammatory markers were also elevated: white blood count (WBC): 21.1/ nl (normal limits 4–12/nl) and c-reactive protein (CRP): 16 mg /dl (normal limits: 0–0.5 mg/dl). Acute cholecystitis was suspected on abdominal ultrasound. A ct scan was ordered to exclude post-interventional complications. Acute cholecystitits was confirmed on ct. Besides, a large volume of gas was found at the FNH site (Fig. 5a & b) without an abscess formation. The patient was placed on intravenous broadband antibiotics and closely monitored. Symptoms and blood chemistry normalized so that the patient was discharged 12 days after TAE. Follow-up continued in out-patient center. The gas formation was completely resorbed 3 weeks later (Fig. 6a & b). Cholecystectomy was performed 6 weeks after TAE. Histopathology of the removed gallbladder showed signs of chronic cholecystitis.

## Discussion and conclusion

Follicular nodular hyperplasia is estimated to constitute close to 8% of all primary hepatic masses and is the second most common benign liver neoplasm after hemangioma [1]. FNH occurs in both sexes and at all ages with a female predominance. Its prevalence has been shown to be high in females on oral contraceptive. FNH in male patients is usually singular, smaller and atypical compared to findings in female patients [1].

In most cases, FNH is clinical silent and is usually diagnosed incidentally during routine abdominal ultrasound. However, clinical symptoms, mostly mild pain or discomfort in the upper abdomen as well as a palpable abdominal mass might be present. The diagnosis is usually suspected following abdominal imaging via US, CT or MRI [3, 10–12]. FNH might be difficult to differentiate from other hepatic lesions based on imaging alone. Lesions like hepatic adenoma and telangiectasia represent the most common differential diagnoses [13]. Such entities, especially hepatic adenoma, a precancerous liver tumor must be differentiated from the benign FNH [1, 2]. This is achieved via histopathology following ultrasound guided fine needle biopsy [12].

Considering the benign natural history of FNH with rare acute complications and lack of malignant potential, asymptomatic cases should be conservatively managed. Surgical resection or interventional management should therefore be reserved for cases with doubtful histology or persistent symptomatic [14, 15].

Transarterial embolization is a well-established procedure in the management of hepatic lesions. TAE was initially used by Doyon and colleagues to manage hepatocellular carcinoma in 1974 [16]. Nowadays, TAE is frequently used for the management of unresectable hepatic tumors. Post-interventional complications especially hepatic failure, intraperitoneal rupture and profuse hemorrhage have been reported in association with TAE [17]. Tissue sublimation however, has so far not been reported as a possible complication of TAE. Herein we report the first case of hepatic tissue sublimation following TAE for a large FNH of the right liver lobe.

The 30 year - old patient presented with upper abdominal pain due to a rapid progressing mass in the right liver lobe. Fine needle biopsy had confirmed the mass to be an FNH. The risk of morbidity following surgical resection was deemed very high for this benign lesion. Thus TAE was favored. This was performed without any peri-procedural complications. Routine ct control on post-interventional day one was within normal limits. Pain to the right upper quadrant with fever and elevated inflammatory markers and liver enzymes prompted imaging via US and ct on day two following TAE. A reduction of the follicular nodular hyperplastic mass was documented on ct scan. Besides, a large gas collection without abscess formation was also seen on ct. The lack of abscess as a possible source of the gas raises the question if sublimation of hepatic tissue had occurred following TAE.

The patient was managed conservatively with intravenous broadband antibiotics, bowel rest and pain medication and was closely monitored with serial ultrasound, blood chemistry and physical examination. No invasive or interventional treatment was warranted due to prompt symptom relief and normalization of both inflammatory markers and hepatic enzymes.

An alternative management could have been surgery or interventional placement of a drain into the gas formation. These options were quickly discarded due to the benign course of this complication. Besides, both alternatives would have created a communication between a potentially aseptic intrahepatic milieu and a septic extra-peritoneal environment thereby increasing the risk of infectious and septic complications.

The mechanism behind this change of state is yet to be understood. The fact that TAE was performed with standardized dosage virtually excludes a dose-dependent complication. Equally, a direct procedure-related explanation for this phenomenon could not be provided.

Gas collection as a sign of abscess formation could have explained this finding. This thesis was improbably for two reasons: first the early onset of gas formation (2 days post TAE) and second the lack of fluid collection (abscess) at TAE site argue against this thesis. However, the good response to broadband antibiotics might argue for an optimally controlled bacterial superinfection by gas building bacteria.

The symptoms: pain to the right upper quadrant, elevated liver enzymes, elevated inflammatory markers in blood and fever leading to imaging on day two after TAE are typical for acute cholecystitis. An alluring hypothesis in this situation would see acute cholecystitis as a sequela of TAE due to the close proximity of the gallbladder to the FNH. This being the case, it remains questionable if this complication would have been discovered in the absence of these symptoms. Nevertheless, the question remains whether or not the symptoms and changes in blood chemistry could have been secondary to TAE or both.

The patient was discharged 12 days after TAE. Follow-up continued at our out-patient services. The gas was completed resorbed in the course of follow – up within 3 weeks. Cholecystectomy was performed 6 weeks after TAE.

Tissue sublimation following TAE has hardly been reported in medical literature. Conservative management with pain medication, bowel resting and broadband antibiotics constitute first line treatment. Surgical or interventional management should be reserved for complicated cases.

## Abbreviations

AP: Alkaline phosphatase
CRP: C- reactive protein
Ct: computed tomography
FNH: Follicular nodular hyperplasia
GGT: Gamma-Glutamyl-Transferase
GOT: Glutamat-Oxalacetat -Transferase
GPT: Glutamat-Pyruvat -Transferase
MRI: Magnet resonance imaging
TAE: Transarterial embolisation
US: Ultrasound

## References

[1] Nguyen BN, Flejou JF, Terris B, Belghiti J, Degott C (1999). Focal nodular hyperplasia of the liver: a comprehensive pathologic study of 305 lesions and recognition of new histologic forms. Am J Surg Pathol.

[2] Vilgrain V, Lewin M, Vons C, Denys A, Valla D, Flejou JF, Belghiti J, Menu Y (1999). Hepatic nodules in Budd-Chiari syndrome: imaging features. Radiology.

[3] Cherqui D, Rahmouni A, Charlotte F, Boulahdour H, Metreau JM, Meignan M, Fagniez PL, Zafrani ES, Mathieu D, Dhumeaux D (1995). Management of focal nodular hyperplasia and hepatocellular adenoma in young women: a series of 41 patients with clinical, radiological, and pathological correlations. Hepatology.

[4] Chen MS, Li JQ, Zheng Y, Guo RP, Liang HH, Zhang YQ, Lin XJ, Lau WY (2006). A prospective randomized trial comparing percutaneous local ablative therapy and partial hepatectomy for small hepatocellular carcinoma. Ann Surg.

[5] Veltri A, Moretto P, Doriguzzi A, Pagano E, Carrara G, Gandini G (2006). Radiofrequency thermal ablation (RFA) after transarterial chemoembolization (TACE) as a combined therapy for unresectable non-early hepatocellular carcinoma (HCC). Eur Radiol.

[6] Buscarini E, Danesino C, Plauchu H, de Fazio C, Olivieri C, Brambilla G, Menozzi F, Reduzzi L, Blotta P, Gazzaniga P (2004). High prevalence of hepatic focal nodular hyperplasia in subjects with hereditary hemorrhagic telangiectasia. Ultrasound Med Biol.

[7] Lewis AL, Adams C, Busby W, Jones SA, Wolfenden LC, Leppard SW, Palmer RR, Small S (2006). Comparative in vitro evaluation of microspherical embolisation agents. J Mater Sci Mater Med.

[8] Lewis AL, Gonzalez MV, Lloyd AW, Hall B, Tang Y, Willis SL, Leppard SW, Wolfenden LC, Palmer RR, Stratford PW (2006). DC bead: in vitro characterization of a drug-delivery device for transarterial chemoembolization. J Vasc Interv Radiol.

[9] Lewis AL, Taylor RR, Hall B, Gonzalez MV, Willis SL, Stratford PW (2006). Pharmacokinetic and safety study of doxorubicin-eluting beads in a porcine model of hepatic arterial embolization. J Vasc Interv Radiol.

[10] Brancatelli G, Federle MP, Grazioli L, Blachar A, Peterson MS, Thaete L (2001). Focal nodular hyperplasia: CT findings with emphasis on multiphasic helical CT in 78 patients. Radiology.

[11] Vilgrain V, Flejou JF, Arrive L, Belghiti J, Najmark D, Menu Y, Zins M, Vullierme MP, Nahum H (1992). Focal nodular hyperplasia of the liver: MR imaging and pathologic correlation in 37 patients. Radiology.

[12] Zins M, Vilgrain V, Gayno S, Rolland Y, Arrive L, Denninger MH, Vullierme MP, Najmark D, Menu Y, Nahum H (1992). US-guided percutaneous liver biopsy with plugging of the needle track: a prospective study in 72 high-risk patients. Radiology.

[13] Brancatelli G, Federle MP, Blachar A, Grazioli L (2001). Hemangioma in the cirrhotic liver: diagnosis and natural history. Radiology.

[14] Nahm CB, Ng K, Lockie P, Samra JS, Hugh TJ (2011). Focal nodular hyperplasia--a review of myths and truths. J Gastrointest Surg.

[15] Perrakis A, Demir R, Muller V, Mulsow J, Aydin U, Alibek S, Hohenberger W, Yedibela S (2012). Management of the focal nodular hyperplasia of the liver: evaluation of the surgical treatment comparing with observation only. Am J Surg.

[16] Doyon D, Mouzon A, Jourde AM, Regensberg C, Frileux C (1974). Hepatic, arterial embolization in patients with malignant liver tumours (author's transl). Ann Radiol (Paris).

[17] Takayasu K, Arii S, Ikai I, Omata M, Okita K, Ichida T, Matsuyama Y, Nakanuma Y, Kojiro M, Makuuchi M (2006). Prospective cohort study of transarterial chemoembolization for unresectable hepatocellular carcinoma in 8510 patients. Gastroenterology.

